# Longitudinal Monitoring of Pan-Immune–Inflammation Value Forecast Outcomes for Patients with Head and Neck Cancer Treated with Chemoradiotherapy or Radiotherapy: Results from a Large Cohort Study

**DOI:** 10.3390/biomedicines14040830

**Published:** 2026-04-05

**Authors:** Sean Hsiang-Ting Chen, Tsung-You Tsai, Rodney Cheng-En Hsieh, Kai-Ping Chang, Chung-Jan Kang, Yi-An Lu, Pei-Wei Huang, Miao-Fen Chen, Chien-Yu Lin, Shanli Ding, Ngan-Ming Tsang, Wen-Hsin Lu, Wing-Keen Yap, Alex Chia-Hsin Lin

**Affiliations:** 1 Department of Radiation Oncology, Linkou Chang Gung Memorial Hospital, Chang Gung University, Taoyuan 33305, Taiwan; sean821016@gmail.com (S.H.-T.C.);; 2 Department of Otorhinolaryngology, Head and Neck Surgery, Chang Gung Memorial Hospital, Medical College of Chang Gung University, Taoyuan 33305, Taiwan; 3 Medical Imaging and Radiological Sciences, Chang Gung Memorial Hospital at Linkou, Chang Gung University, Taoyuan 33305, Taiwan; 4 Institute of Stem Cell and Translational Cancer Research, Chang Gung Memorial Hospital at Linkou, Chang Gung University, Taoyuan 33305, Taiwan; 5 Institute of Immunology and Translational Medicine, Chang Gung University, Taoyuan 33305, Taiwan; 6 Department of Hematology-Oncology, Chang Gung Memorial Hospital, Medical College of Chang Gung University, Taoyuan 33305, Taiwan; 7 Department of Radiation Oncology, Chiayi Chang Gung Memorial Hospital, Chiayi 613016, Taiwan; 8 Graduate School of Biomedical Sciences, The University of MD Anderson Cancer Center, Houston, TX 77030, USA; 9 Department of Radiation Oncology, China Medical University Hsinchu Hospital, Zhubei 30272, Taiwan; 10 Departments of Family Medicine and Occupational Medicine, Taipei City Hospital, Zhong-Xiao Branch, Taipei 11556, Taiwan

**Keywords:** pan-immune–inflammation value, head and neck cancer, radiotherapy, biomarker, longitudinal monitoring

## Abstract

**Background/Objectives**: We aim to investigate whether tracking pan-immune–inflammation value (PIV) dynamics during radiotherapy (RT) can inform real-time prognosis in patients with head and neck cancer (HNC). **Methods**: We retrospectively reviewed the medical records of patients with HNC who received RT at our institution between 2005 and 2013. Temporal changes in the PIV throughout the RT were evaluated using the Friedman test and Wilcoxon signed-rank test. The PIV dynamics were quantified using PIV ratios, defined as the PIV at three distinct time points (PIV-2, PIV-4, and PIV-6) during treatment divided by the pretreatment PIV (PIV-0). Overall survival (OS) and progression-free survival (PFS) served as the primary and secondary endpoints analyzed. **Results**: A total of 676 patients with HNC were enrolled, with a median follow-up of 8.1 years. The PIV demonstrated a continuously ascending trend over time, with the most dramatic increase occurring six weeks after the start of RT. Compared with patients with a low PIV ratio at six weeks (PIV-6/PIV-0), those with a high PIV ratio showed more favorable survival outcomes (five-year OS: 58.9% versus 70.8%, *p* = 0.002; five-year PFS: 62.0% versus 71.1%, *p* = 0.013). The subgroup analyses yielded consistent results. Notably, the real-time risks of death and recurrence changed in parallel with the PIV dynamics. Multivariate analysis confirmed PIV-6/PIV-0 as an independent prognostic factor for both OS and PFS. **Conclusions**: Monitoring longitudinal PIV dynamics may assist in forecasting the OS and PFS in patients with HNC being treated with RT, thus enabling individualized, risk-adapted treatment management.

## 1. Introduction

The global incidence and mortality of head and neck cancer (HNC) have increased considerably over the past decade, with approximately 500,000 new cases and 270,000 deaths reported annually [1,2]. This upward trend poses a formidable challenge to public health worldwide. The multidisciplinary strategy that combines radiotherapy (RT) and platinum-based chemotherapy has significantly improved patients’ overall survival (OS) and has enhanced functional organ preservation, with manageable toxicity [3,4,5,6,7]. However, the survival benefits of these interventions are not consistently observed, with studies suggesting the existence of strong inter-patient heterogeneity. For instance, studies have recorded wide ranging figures in terms of 3-year OS (67–91%) and progression-free survival (PFS) (79–81%) in patients with nasopharyngeal cancer (NPC) [8,9,10,11,12,13,14], while pharyngo-laryngeal cancer cases displayed an absolute interstudy difference of 10% for 2-year OS and 15% for PFS [15,16,17,18]. Although the UICC TNM staging system remains a cornerstone of cancer prognostication, its primary focus on anatomical features cannot fully account for the broader biological heterogeneity influencing clinical outcomes [19,20]. Moreover, the risks of death and disease progression are not static, but fluctuate throughout the treatment course [21,22,23]. This situation highlights the need to identify novel real-time biomarkers that facilitate dynamic risk assessments for individualized treatment adaptations, either towards intensification or de-escalation.

Mounting evidence suggests that systemic immunity and inflammation play a pivotal role in tumor evolution [24,25]. These biological processes can be mirrored in systemic/circulating blood counts, making them attractive prognostic biomarkers [22,26,27,28,29]. Specifically, circulating myeloid cells, such as neutrophils and monocytes, can infiltrate the tumor, exerting activity, including the promotion of cancer cell proliferation, metastasis, angiogenesis, and immunosuppression [30,31]. Additionally, circulating platelets can shield tumor cells from immune detection and facilitate their entrapment at the endothelium of distant sites [32]. In contrast, lymphocytes, which comprise one-third of the total number of white blood cells, are recruited from the periphery to the tumor site, where they recognize and eliminate neoplastic cells [33,34,35]. Altogether, these hematological cells work in concert with RT and chemotherapy, modulating their efficacy and have a significant influence on patient outcomes [36,37].

Building upon this foundation, Fuca et al. were the first to propose a novel index, the pan-immune–inflammation value (PIV), which integrates these four circulating blood cell types, that demonstrated robust capacity for predicting survival outcomes in metastatic colorectal cancer patients [38,39]. The PIV formula consists of a numerator (neutrophils × platelets × monocytes) and a denominator (lymphocytes). This reflects the ratio of circulating pro-inflammatory cells to lymphocytes, directly capturing the interplay between systemic inflammation and anti-tumor immunity [29,40]. Since then, the clinical utility of the PIV has been extended to various cancers, including gastro-intestinal, lung, genitourinary, and HNC [28,29,40,41,42,43,44]. However, the majority of existing studies have primarily focused on the pretreatment PIV. There is a lack of detailed characterization of PIV dynamics, particularly during RT [45]. This research gap is noteworthy, as RT and chemotherapy are well-recognized as being responsible for inducing alterations in circulating blood cell counts. This also begs the question as to whether temporal changes in the PIV during treatment could have prognostic utility in HNC. Therefore, this study aims to specifically address these issues.

## 2. Materials and Methods

### 2.1. Patient Selection

This retrospective study reviewed the clinical records of 1431 patients diagnosed with squamous cell carcinomas of the head and neck between 2005 and 2013. After excluding individuals lacking peripheral blood count data at the baseline, as well as at two, four, or six weeks following the initiation of RT, a total of 676 patients were enrolled in our study (Supplement Figure S1). All of the patients received primary treatment with either RT or platinum-, cetuximab-, or Taxotere-based concurrent chemoradiotherapy (CCRT) at Linkou Chang Gung Memorial Hospital, a tertiary medical center in Taiwan. Eligible tumors sites included nasopharyngeal, oropharyngeal, hypopharyngeal, and laryngeal carcinomas. Immunotherapy was not routinely administered as a primary treatment during the study period. Pretreatment staging was based on the American Joint Committee on Cancer (AJCC) Staging Manual, seventh edition. The exclusion criteria comprised of the following: (1) age below 18 years, (2) treatment duration exceeding 70 days, (3) primary treatment involving surgery, (4) RT dose expressed as equivalent dose in 2 Gy fractions (EQD2) less than 66.0 Gy, (5) diagnosis of a second primary cancer within three years preceding or following treatment, and (6) the detection of distant metastasis before the completion of the primary treatment. The research protocol adhered to the principles in the Helsinki Declaration and received ethical approval from the local Institutional Review Board (approval number: 202000190B0). Due to the retrospective nature of the study, the requirement for informed consent was waived.

### 2.2. Data Collection and Outcome Measures

Radiation oncologists and experienced nurses conducted independent reviews of the clinical records, gathering the following variables of interest: age, gender, comorbidities, tumor location, clinical stage, duration of RT, prescribed radiation dose (EQD2), RT technique (including intensity-modulated radiation therapy (IMRT) and volumetric modulated arc therapy (VMAT)), smoking history, alcohol consumption, betel quid chewing behavior, and occurrence of death, along with disease progression. The clinical data were extracted from the comprehensive cancer registry and the meticulous electronic medical records held at our institution. There were no missing data in regard to the baseline characteristics and covariates present in the analyzed cohort. In accordance with the standard care protocol of our institution, all patients underwent weekly outpatient reviews carried out by radiation oncologists during their radiotherapy course. Complete blood counts were routinely monitored to assess acute treatment-related toxicities (e.g., infection, mucositis, or malnutrition) or potential hematopoietic suppression arising from irradiation. According to our institutional guidelines, cisplatin-based chemotherapy is the preferred regimen for concurrent administration during RT. For patients who are ineligible for cisplatin, carboplatin or cetuximab is recommended as an alternative. The two main concurrent chemotherapy regimens utilized in this study: (1) intravenous cisplatin (50 mg/m^2^) combined with oral tegafur with or without leucovorin for 14 days and (2) intravenous cisplatin (30–40 mg/m^2^) administered weekly. Dose modifications were implemented based on the patient’s hematologic tolerance and renal function. Complete blood counts (CBCs) were collected for all patients within two weeks prior to RT, as well as at two, four, and six weeks after the commencement of RT. The following data were derived from the CBCs, including the neutrophil, lymphocyte, monocyte, and platelet counts. The pan-immune–inflammation value (PIV) was calculated by multiplying the neutrophil, platelet, and monocyte counts, which was then divided by the lymphocyte count. The primary endpoint was overall survival (OS), which was defined as the duration from the initial day of RT until the patient’s death or censoring (the date of their last follow-up). The secondary endpoint was progression-free survival (PFS), which was defined as the time interval from the first day of the patient’s RT to the date of disease progression, death, or censoring.

### 2.3. Variable Definitions

The pretreatment PIV was labeled as PIV-0, with PIV-2, PIV-4, and PIV-6 corresponding to the PIV values at two, four, and six weeks after the beginning of RT, respectively. To depict the dynamics of the on-treatment PIV, we designed a novel index, the PIV ratio, which is calculated by dividing the PIV during treatment (PIV-2, PIV-4, and PIV-6) by the pretreatment PIV (PIV-0). The treatment duration, defined as the timeframe from the first to the last day of RT, was categorized as >56 days versus ≤56 days, based on the cut-off proposed previously in the literature [46]. Comorbidities were classified as yes versus no, based on the Charlson Comorbidity Index [47]. Risky lifestyles, including cigarette smoking (current or former smokers versus non-smokers), alcohol consumption (current or former drinkers versus non-drinkers), and betel quid chewing behavior (current or former chewers versus non-chewers), were similarly categorized as yes versus no.

### 2.4. Data Analysis

Continuous variables were expressed as means (standard deviations) and medians (interquartile ranges; IQRs). Categorical variables were represented as counts and percentages. The Friedman test was conducted to evaluate the overall evolution of the PIV across all time points, and the Wilcoxon signed-rank test with Bonferroni correction was performed for post hoc pairwise comparisons between individual time points. The optimal cut-off values for the PIV ratios at different time_points were determined using Youden’s index, based on receiver operating characteristic (ROC) curve analysis. Time-to-event analyses were conducted using the Kaplan–Meier method and comparisons were made using the log-rank test. Harrell’s Concordance Index (C-index) was utilized to evaluate the discriminative ability of thePIV ratio. The proportional hazards assumption for the Cox regression models was verified using a scaled Schoenfeld residuals plot (Supplement Figure S2). Factors demonstrating significant associations (*p* < 0.05) in the univariable analysis were included as covariates in the multivariable Cox models, with the Enter method used to identify independent predictors of the study outcomes. Independent predictors identified in the univariable analysis were internally validated using the bootstrapping method (1000 repetitions) and discriminative model performance was evaluated using the Apparent and Optimism-Corrected Concordance Index (C-index). The results are presented as hazard ratios (HRs), with corresponding 95% confidence intervals. All calculations and visualizations were performed using MedCalc Statistical Software version 23.0.2 (MedCalc Software Ltd., Ostend, Belgium; https://www.medcalc.org (accessed on 28 August 2024) and RStudio (version 2026.01.1). A two-tailed *p*-value of less than 0.05 was considered statistically significant.

## 3. Results

### 3.1. Baseline Characteristics

The demographic and clinical characteristics of 676 head and neck carcinoma patients are summarized in Table 1. The median age was 50.4 years (IQR: 44.2–57.8 years), and the majority of the patients were male (86.0%). Of the 676 patients, 57.5% were diagnosed with locally advanced disease (T3–4), and 52.7% had N2–3 disease. The nasopharynx was the most common primary tumor site (50.0%), followed by the oropharynx (23.1%), hypopharynx (20.1%), and larynx (6.8%). Most of the study patients received CCRT (85.1%). Regarding the radiotherapy techniques used, the cohort was evenly distributed in regard to IMRT (50.4%) and VMAT (49.6%). The majority of patients (96.0%) received concurrent cisplatin-based regimens (Supplement Table S1). All participants completed the planned course of RT, with a median duration of 52 days (IQR: 51–55 days) and a median EQD2 of 72 Gy (IQR: 72–72 Gy).

As detailed in Supplement Table S2, the NPC subgroup exhibited characteristics typical of an endemic population in Taiwan, with a high prevalence of WHO Type III histology (91.1%), a male predominance (79.0%), and a balanced distribution of advanced tumor burden (T3–4: 51.2%; N2–3: 48.2%). The treatment protocols were highly consistent, with 93.8% of patients receiving cisplatin-based chemotherapy and a comparable utilization of IMRT (47.9%) versus VMAT (52.1%).

### 3.2. PIV Dynamics During RT

**Figure 1:**
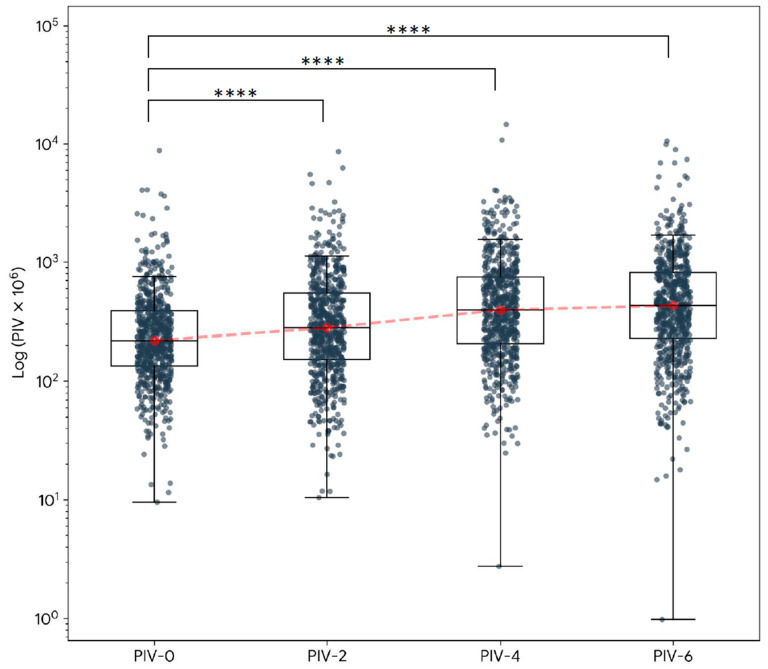
Longitudinal course of pan-immune–inflammation values in patients with head and neck cancer being treated with radiotherapy. Pan-immune–inflammation values were measured during the pretreatment phase (PIV-0) and at two (PIV-2), four (PIV-4), and six (PIV-6) weeks after the start of radiotherapy. Wilcoxon signed-rank test was used to compare the differences between two time points (i.e., from pre-RT to measures acquired during RT). All individual data points, including extreme outliers, are visualized as background scatter points. The red dashed line connects the median values across the treatment time points. The central horizontal line represents the median, and the upper and lower bounds indicate the 75th and 25th percentiles in the box plot. **** *p* < 0.0001.

The longitudinal changes in the PIV during treatment are illustrated in Figure 1 and Table 2. The PIV showed a continuously ascending trend over time (*p* < 0.0001, Friedman test), with median values rising from 218.9 at the baseline (PIV-0) to 278.1 at two weeks (PIV-2), 393.7 at four weeks (PIV-4), and 428.6 at six weeks (PIV-6) following the start of RT. The post hoc analysis confirmed significant differences between the PIV at each time point during RT (PIV-2, PIV-4, and PIV-6) and the baseline (PIV-0) (*p* < 0.0001, adjusted with Bonferroni correction). Of note, the most dramatic increase occurred at six weeks after the beginning of RT, with the median PIV ratio (PIV-6/PIV-0) increasing to 1.78 (IQR: 0.8–4.3).

### 3.3. Survival Analysis

**Figure 2:**
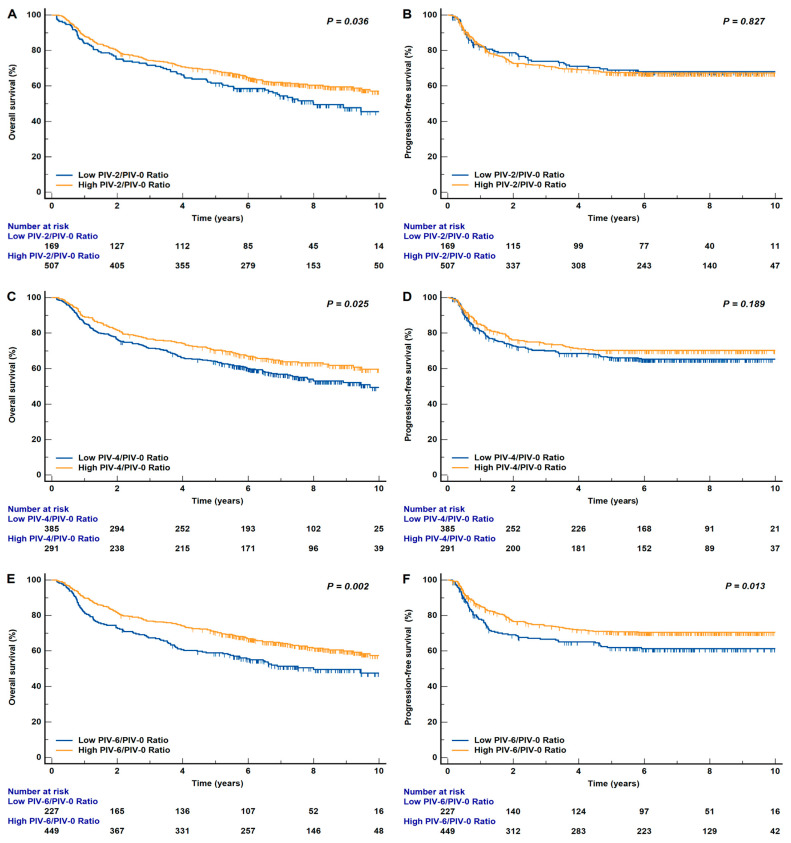
Kaplan–Meier plots of OS and PFS according to the PIV ratios at two weeks (**A**,**B**), four weeks (**C**,**D**), and six weeks (**E**,**F**) after the start of radiotherapy in the entire cohort (*n* = 676).

**Figure 3:**
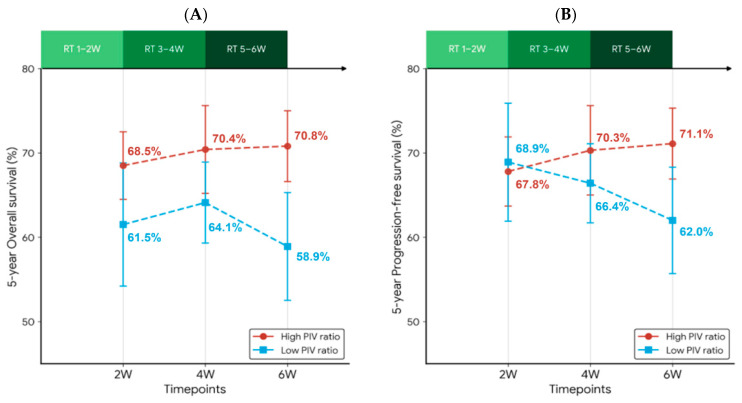
Synchronous changing map of the 5-year OS (**A**) and PFS (**B**) with on-treatment PIV ratios at two weeks, four weeks, and six weeks during RT. At each time point, the patients were stratified into high (red circles) and low (blue squares) PIV ratio groups. The numerical values labeled next to each marker represent the specific 5-year survival percentages. The green bar at the top illustrates the progressive timeline of the RT course, divided into two-week intervals (RT 1-2W, RT 3-4W, and RT 5-6W). The 5-year OS and PFS rates were calculated using the Kaplan–Meier method and the vertical error bars indicate the corresponding 95% confidence intervals. The dashed lines highlight the diverging trajectories, illustrating the increasing prognostic discriminative power of the PIV ratio during RT.

Patients were then categorized into low and high-risk groups based on the optimal cut-off values derived from the ROC curve, using the Youden index, for assessing the risk of death. The optimal cut-off values were identified as 0.76, 1.13, and 1.11 for PIV-2/PIV-0, PIV-4/PIV-0, and PIV-6/PIV-0, respectively. The median follow-up duration was 8.10 years. Compared with patients with a low PIV ratio at six weeks, those with a high PIV ratio showed more favorable survival outcomes (five-year OS: 58.9% versus 70.8%, respectively, *p* = 0.002; five-year PFS: 62.0% versus 71.1%, respectively, *p* = 0.013; Figure 2). Similar findings were observed for the patients’ OS when the PIV ratio was measured at two weeks (five-year OS: 61.5% versus 68.5%, respectively, *p* = 0.036) and at four weeks (five-year OS: 64.1% versus 70.4%, respectively, *p* = 0.025; Figure 2). In contrast, neither PIV4/PIV0 nor PIV2/PIV0 could significantly stratify the patients in terms of PFS (*p* = 0.189 and *p* = 0.827, respectively). Moreover, we illustrated the five-year OS and PFS alongside the dynamic PIV ratios over_time and observed that the patients’ real-time risks of death or recurrence synchronously changed with the PIV dynamics during RT. Specifically, the mid-treatment PIV ratio served as a dynamic biomarker that refined the prognosis. While the survival estimates between the high and low PIV ratio groups were relatively proximate at week 2, a substantial and significant divergence emerged by week 6. This widening survival discrimination in regard to OS and PFS between the high and low PIV ratios became increasingly evident at later treatment time points (Figure 3A–B).

Next, we conducted subgroup analyses to examine the potential impact of selection bias on the prognostic importance of the PIV ratio. First, we restricted our analysis to patients with NPC, the most prevalent subtype of HNC in our study population. The results largely recapitulated those observed within the entire cohort, the only exception being the association of the PIV ratio at week 6 with PFS (Supplement Figure S3). Second, we repeated this analysis for patients with oropharyngeal cancer, the second most common subtype of HNC in our study population. The results consistently supported the association between the PIV ratio at week 6 and OS (Supplement Figure S4). Third, the analysis was limited to patients with locally advanced disease (i.e., T3–4). Likewise, the results were found to be consistent, with the exception of a lack of association between the PIV ratio at week 4 and OS (Supplement Figure S5). Fourth, a subgroup analysis of patients with N2–N3 disease revealed comparable findings, substantiating the association between the PIV ratio at weeks 4 and 6 and OS, as well as between the PIV ratio at week 6 and PFS (Supplement Figure S6).

Significant univariable prognostic factors (*p* < 0.05) for the primary endpoint (OS) and the secondary endpoint (PFS) were subsequently incorporated into multivariable Cox regression models. The clinical variables that were independently associated with OS in the multivariable analysis included age, the advanced tumoral as well as the nodal stage, primary tumor site, existing comorbidities, and RT technique. With respect to the PIV dynamics-derived parameters, a higher PIV ratio at six weeks (PIV-6/PIV-0) stood out as an independent predictor of improved OS (HR: 0.68; 95% CI: 0.52–0.90; *p* = 0.006), whereas PIV4/PIV0 and PIV2/PIV0 did not retain prognostic significance in the multivariate analysis (Table 3).

The clinical variables that were independently associated with PFS in the multivariable analysis were the tumor stage, nodal stage, primary tumor site, alcohol consumption, and RT technique. In line with the findings for OS, PIV-6/PIV-0 emerged as a significant independent factor for PFS (HR: 0.68; 95% CI: 0.51–0.91; *p* = 0.008), while PIV4/PIV0 and PIV2/PIV0 did not hold prognostic significance after accounting for confounding variables (Table 3), suggesting the unique prognostic importance of PIV dynamics measured at later time points during treatment.

To assess the discriminative ability of the PIV-6/PIV-0 ratio, we first evaluated it as a dichotomized variable in the univariate analysis, yielding a Harrell’s C-index of 0.548 (95% CI: 0.519–0.577) for OS and 0.543 (95% CI: 0.509–0.576) for PFS. In the multivariable models, the addition of the PIV-6/PIV-0 ratio provided incremental prognostic value beyond the clinical variables alone. For OS, the C-index improved from 0.703 (95% CI: 0.673–0.733) in the clinical model to 0.717 (95% CI: 0.682–0.741) after the inclusion of the PIV ratio. Similarly, for PFS, the C-index increased from 0.674 (95% CI: 0.637–0.711) to 0.680 (95% CI: 0.642–0.717). These findings indicate that the PIV-6/PIV-0 ratio contributes modest but meaningful incremental discrimination. Subsequently, to assess the robustness of our multivariable models, we performed internal validation using 1000 bootstrap resamples. For OS, the model achieved an apparent C-index of 0.717, with an optimism-corrected C-index of 0.699 (optimism = 0.018). Similarly, for PFS, the apparent C-index of 0.680 decreased minimally to 0.660 after correction (optimism = 0.020), supporting the stability of the model.

## 4. Discussion

In this long-term follow-up involving a large-scale cohort, we retrospectively enrolled 676 patients with HNC and systematically documented their circulating blood cell counts at four distinct time points before and throughout the whole course of RT. Notably, we observed a consistent increase in the PIV during treatment, and this trend was significantly associated with favorable survival outcomes. Further analysis of the on-treatment PIV dynamics identified the late phase PIV ratio (PIV-6/PIV-0) as a robust and independent prognostic marker for both OS and PFS. If externally validated, these findings may establish the PIV as a cost-effective and readily available biomarker during RT in patients with HNC, offering a promising avenue for the development of risk-adapted therapeutic strategies.

Previous PIV studies in patients with HNC have primarily focused on pretreatment values, which captured only a static snapshot of the baseline status, rather than incorporating multiple time points to capture its trajectories during treatment [28,29,43]. Other than HNC, a few studies have examined the temporal changes in the PIV and its impact on survival outcomes, as well as the treatment response. In patients with advanced non-small-cell lung cancer (NSCLC), Chen et al. demonstrated that low PIV dynamics (assessed at weeks 3–4 after immunotherapy initiation compared to the baseline) were significantly correlated with prolonged PFS and OS, as well as a higher incidence of immune-related adverse events [48]. Similar findings were reported by Corti et al. in patients with microsatellite instability-high metastatic colorectal cancer (CRC), where an increase (≥30%) in the PIV within 3–4 weeks after immune checkpoint inhibitor initiation was linked to poorer treatment responses and inferior OS and PFS [49]. Interestingly, Perez-Martelo et al. found that early increases in the PIV were not associated with survival or disease control, whereas elevations observed at later treatment time points (beyond 4 weeks post-treatment initiation) correlated with disease progression [50]. Collectively, our findings are in line with previous investigations indicating that PIV trajectories during treatment are prognostically informative, with the most pronounced impacts observed during the later stage of therapy.

However, previous studies were distinct from ours in terms of both their design and scope, and several limitations remain unaddressed. First, prior research efforts were conducted in regard to other solid tumor types and predominantly examined patients with metastatic disease treated with systemic therapies (i.e., metastatic CRC patients receiving chemotherapy; NSCLC patients undergoing immunotherapy), thereby limiting the generalizability of the findings to patients with non-metastatic or early-stage diseases [48,50]. In contrast, our study exclusively focused on patients with non-metastatic HNC managed with definitive local RT or CCRT, adding credence to the prognostic utility of PIV dynamics during locoregional treatment with curative intent. Second, the suboptimal statistical power of previous studies, attributable to limited cohort sizes (i.e., fewer than 300 cases) and relatively short follow-up durations (i.e., less than 3 years), hindered their capacity to offer a robust foundation for the longitudinal analysis of patient survival [48,49]. Finally, prior investigations examined the PIV at only a single time point during therapy (i.e., 3–4 weeks after treatment initiation), which precludes any further understanding of the temporal complexity of PIV dynamics, especially in regard to the prolonged duration of treatment [49,50]. In contrast, we have provided, so far, the most exhaustive on-treatment PIV data gathered throughout the whole-course of RT.

To contextualize these prognostic findings within the biological complexity of the disease, it is important to note that HNSCC accounts for approximately 90% of cases. Despite a uniform histological classification, gene expression profiling has identified at least six molecular subtypes with distinct immune and inflammatory signatures and divergent clinical behaviors [51]. Notably, nearly 40% of HNSCC cases belong to an ‘Immune Class,’ which can be further divided into two phenotypes: an ‘Active’ subtype characterized by M1 macrophage enrichment and favorable outcomes, and an ‘Exhausted’ subtype associated with M2 macrophage infiltration, TGF-β/WNT signaling, and inferior survival [52]. Viral etiology further contributes to this heterogeneity. HPV-positive HNSCC exhibits highly inflamed tumor microenvironments (TMEs) enriched with CD4+ and CD8+ T cells, B cells, and plasma cells, corresponding to a ‘lymphocyte-rich’ phenotype and improved prognosis [53,54]. In contrast, HPV-negative HNSCC, often linked to tobacco exposure, is characterized by NF-κB-driven inflammatory signaling and a more immunosuppressive profile [55,56]. Beyond HNSCC, NPC represents a biologically distinct entity. Specifically, the Epstein–Barr virus (EBV)-associated, non-keratinizing subtype develops within a chronically inflamed, lymphocyte-rich TME, with prominent interferon signaling [57,58]. Collectively, these diverse subtypes contribute to substantial variability in both the local TMEs and systemic inflammatory states across HNC.

Although RT is widely recognized as a locoregional treatment for cancers, growing evidence has indicated its systemic immunologic and inflammatory effects beyond the irradiated field [37,59,60]. For example, RT has been shown to induce neutrophil recruitment into the tumor microenvironment, where they suppress T cell proliferation and function, thereby promoting tumor progression [61]. Moreover, declines in peripheral neutrophil counts after RT were associated with treatment response and survival outcomes in cervical and rectal cancers [61,62]. Similarly, RT-induced platelet dynamics have also been observed in oropharyngeal cancer, characterized by a biphasic pattern, an initial decrease during the acute phase of RT, followed by a compensatory post-treatment increase. Moreover, persistently elevated platelets at 6- and 12-months post-RT were correlated with inferior OS [32,36]. Rather than relying solely on individual blood cell components, integrated indices, such as the lymphocyte-to-monocyte ratio (LMR), have also garnered much attention. Interestingly, RT-driven increases in the LMR have been shown to correlate with favorable disease control and OS in both post-operative RT and definitive RT settings [22,63,64]. Based on these findings, it is hypothesized that PIV dynamics can capture the complex interplay between and temporal changes of these four circulating blood cells simultaneously.

While a high pretreatment PIV is conventionally associated with a poor prognosis, an elevated PIV ratio during RT may reflect a distinct biological context. Rather than conferring negative prognostic significance, a higher on-treatment PIV ratio may indicate effective host immune activation against the tumor. First, although an increase in the PIV during treatment partly reflects RT-induced lymphopenia [65], RT also induces tumor DNA damage and cell death, prompting the release of tumor-associated antigens and DAMPs (e.g., HMGB1 and calreticulin). This initiates an acute-phase inflammatory response that mobilizes immune cells from the bone marrow into the peripheral circulation [66,67]. Therefore, a high PIV ratio during treatment mirrors not only lymphocyte depletion, but also systemic tumor destruction and active immune mobilization in the host. Second, while a high pretreatment PIV typically represents chronic immunosuppression driven by tumor-secreted cytokines (e.g., via myeloid-derived suppressor cells), the inflammatory flare during radiotherapy represents an acute and transient phase [68,69]. Consequently, the biological context of a high PIV during treatment is fundamentally different from that at the baseline. Third, a therapy-induced PIV surge suggests that the host’s immune system remains highly competent and capable of mounting a vigorous physiological response against the irradiated tumor. Patients lacking this dynamic response (i.e., those with a low PIV ratio) may harbor a relatively exhausted immune system that fails to respond effectively to treatment. Together, these findings support the use of the PIV ratio as an integrative biomarker that is capable of capturing a more comprehensive view of the evolving immunological landscape during RT, which in turn has additional prognostic value.

## 5. Limitations

There are some limitations to this study. First, it was conducted retrospectively at a single institution, which may introduce selection bias. Nevertheless, as the largest tertiary referral center in Taiwan, this concern is likely mitigated by the fact that our institution receives referrals or draws patients from diverse geographic and clinical backgrounds across the country. In addition, the exclusion of a substantial portion of the initial population due to missing CBC data may introduce potential immortal time and guarantee-time biases. However, the comparable 90-day early mortality (1.2% vs. 1.1%) and overall survival between the included and excluded patients suggest that the patients excluded due to missing CBC data was not driven by early clinical deterioration or worse prognosis. In addition, the landmark analysis using the end of RT as the starting time yielded consistent results. Furthermore, although we restricted our cohort to patients who completed their full course of RT to ensure uniform treatment exposure, this may limit the study’s generalizability to patients who discontinue treatment early. Further prospective multicenter studies are still warranted to validate the prognostic significance of serial PIV measurements. Second, although we identified a relationship between the PIV ratio and clinical prognosis and proposed possible hypotheses (e.g., RT-induced host immune activation), the mechanistic basis for this association remains to be elucidated. Closing this gap will require comprehensive studies involving the direct profiling of the tumor microenvironment, particularly the composition and function of immune subsets within tumor-infiltrating populations, including cytotoxic CD8+ T-cells, regulatory T-cells (Tregs), or expression of immune checkpoints (e.g., PD-1/PD-L1). Third, since our study was conducted in an NPC-endemic area, over 96% of our NPC subgroup had non-keratinizing carcinoma (WHO Type II/III). Due to this limited variation, we could not perform a meaningful statistical comparison between histological subtypes. However, focusing on this highly homogeneous EBV-associated subtype provides a clearer evaluation of the PIV’s prognostic role specifically within the predominant form of NPC found in endemic regions. Fourth, due to the retrospective nature of this study that spanned a historical period (2005–2013), routine p16 immunohistochemistry for all oropharyngeal cancer (OPC) and quantitative plasma EBV DNA for all nasopharyngeal carcinoma (NPC) were not universally available or standardized in our early-cohort records. In addition, we did not obtain the degree of differentiation in terms of SCC between the subgroups. Consequently, the lack of virus markers and tumor differentiation limited our ability to perform more granular subgroup analyses of those aspects that will influence prognosis in head and neck cancers. Fifth, given that the vast majority of patients received cisplatin-based regimens, we did not perform a differential analysis regarding the impact of different chemotherapy types, dosing schedules (e.g., bi-weekly versus weekly protocols), or regimen-specific toxicities, which inherently limits the generalizability of our results to patients treated with different chemotherapy regimens or dosing strategies. Sixth, we did not collect and assess the post-treatment PIV dynamics, an aspect that may reflect the resilience or sustained dysregulation of circulating immune cells following RT. As a result, we were unable to compare the post-treatment dynamics with the on-treatment PIV changes observed, leaving the potential prognostic value of the post-treatment PIV unexplored. Seventh, this study did not compare the dynamic PIV ratio with other established inflammatory markers (e.g., NLR and LMR). Future studies incorporating multiple biomarkers are warranted to develop more comprehensive predictive models. Finally, as no standardized method currently exists for determining optimal PIV ratio cut-offs and the thresholds were only internally validated in our cohort, these chosen cut-off values must be further evaluated and validated in a well-designed, large-scale prospective study.

## 6. Conclusions

In summary, our study highlights the prognostic significance of longitudinal monitoring of the PIV during RT, with the late-phase PIV ratio emerging as a strong and independent predictor of both OS and PFS in patients with HNC. Since patients with similar baseline characteristics often exhibit divergent outcomes, monitoring the on-treatment dynamic PIV effectively refines the initial prognosis to identify true high-risk subgroups. This complementary risk stratification enables the adoption of a more individualized and cost-effective approach, guiding post-treatment surveillance and timely interventions. If further validated in prospective studies, the dynamic PIV ratio could be leveraged as an affordable and scalable biomarker for real-time risk stratification and tailored treatment adaptation.

## Figures and Tables

**Table 1:** Baseline characteristics of patients with head and neck carcinoma (*n* = 676).

Characteristics	Value	Percentage
Median age, years (IQR)	50.4 (44.2–57.8)	
Male sex	581	86.0
AJCC 7th edition T stage		
T1–2	287	42.5
T3–4	389	57.5
AJCC 7th edition N stage		
N0–1	320	47.3
N2–3	356	52.7
Primary tumor site		
Nasopharynx	338	50.0
Oropharynx	156	23.1
Hypopharynx	136	20.1
Larynx	46	6.8
Cigarette smoking		
Yes	505	74.7
No	171	25.3
Betel quid chewing		
Yes	324	47.9
No	352	52.1
Alcohol consumption		
Yes	312	46.2
No	364	53.8
Presence of medical comorbidities		
Yes	342	50.6
No	334	49.4
Chemotherapy		
Yes	575	85.1
No	101	14.9
Radiotherapy technique		
VMAT	335	49.6
IMRT	341	50.4
Median treatment duration, days (IQR)	52 (51–55)	
Median EQD2, Gy (IQR)	72 (72–72)	

Data are given as counts and percentages, unless otherwise indicated. There were no missing data for the baseline characteristics and covariates present in the analyzed cohort. Abbreviations: AJCC, American Joint Committee on Cancer; EQD2, equivalent dose in 2 Gy fractions; IQR, inter-quartile range; IMRT, intensity-modulated radiation therapy; VMAT, volumetric modulated arc therapy.

**Table 2:** Changes to the pan-immune–inflammation value pre- and during radiotherapy in patients with head and neck cancer (*n* = 676).

	Mean	SD	Median	△Median	IQR	Ratio (During RT/Pre-RT)		*p* Value
						Median	IQR	
PIV, ×10^6^								<0.0001 ^†^
Pretreatment (PIV-0)	352.5	535.9	218.9		133.3–386.6			
During treatment								
2 weeks (PIV-2)	472.4	670.6	278.1	59.2	151.8–547.4	1.17	0.8–2.1	<0.0001 *
4 weeks (PIV-4)	645.3	924.8	393.7	174.8	206.4–751.0	1.78	0.9–3.3	<0.0001 *
6 weeks (PIV-6)	695.9	993.6	428.6	209.7	227.4–818.8	1.78	0.8–4.3	<0.0001 *

Parameters are presented as mean, standard deviation, median, interquartile range, and ratio. The rate of change (△Median) was defined as the absolute difference between the median PIV at each time point (PIV-2, -4, and -6) and the pretreatment median PIV-0. Abbreviations: IQR, interquartile range; PIV, pan-immune–inflammation value; RT, radiotherapy; SD, standard deviation. ^†^ Friedman test was used to assess the overall change in PIV across all time points. * Changes between two timepoints (i.e., from pre-RT to measures acquired during RT) were assessed using Wilcoxon signed-rank test with a Bonferroni correction.

**Table 3:** Univariate and multivariate analyses of prognostic factors for OS and PFS.

	OS				PFS			
	Univariate Analysis		Multivariate Analysis		Univariate Analysis		Multivariate Analysis	
Variable	HR (95% CI)	*p* Value	HR (95% CI)	*p* Value ^c^	HR (95% CI)	*p* Value	HR (95% CI)	*p* Value ^c^
PIV-6/PIV-0 (≥1.11 vs. <1.11) ^a^	0.68 (0.54–0.87)	0.002	0.68 (0.52–0.90)	0.006	0.70 (0.53–0.93)	0.013	0.68 (0.51–0.91)	0.008
PIV-4/PIV-0 (≥1.13 vs. <1.13) ^a^	0.76 (0.60–0.97)	0.025	0.92 (0.70–1.20)	0.529	0.83 (0.63–1.10)	0.190		
PIV-2/PIV-0 (≥0.76 vs. <0.76) ^a^	0.76 (0.59–0.98)	0.036	0.85 (0.65–1.12)	0.256	1.04 (0.75–1.43)	0.827		
Age ^b^	1.03 (1.02–1.04)	<0.001	1.02 (1.01–1.03)	0.004	1.00 (0.99–1.01)	0.680		
Sex (male vs. female)	1.98 (1.30–3.01)	0.001	1.19 (0.74–1.89)	0.477	1.29 (0.85–1.96)	0.235		
AJCC 7th T stage (T3–4 vs. T1–2)	2.07 (1.60–2.67)	<0.001	1.94 (1.49–2.51)	<0.001	1.52 (1.14–2.02)	0.004	1.47 (1.10–1.97)	0.010
AJCC 7th N stage (N2–3 vs. N0–1)	1.60 (1.26–2.03)	0.001	1.58 (1.23–2.03)	<0.001	2.05 (1.53–2.73)	<0.001	1.93 (1.44–2.59)	<0.001
Primary tumor site		0.001		0.002		<0.001		0.061
Oropharynx vs. nasopharynx	2.16 (1.60–2.90)	<0.001	1.72 (1.25–2.37)	<0.001	1.83 (1.30–2.56)	<0.001	1.51 (1.06–2.15)	0.022
Hypopharynx vs. nasopharynx	2.96 (2.21–3.97)	<0.001	1.79 (1.29–2.49)	<0.001	2.04 (1.43–2.89)	<0.001	1.41 (0.97–2.06)	0.076
Larynx vs. nasopharynx	2.01 (1.27–3.20)	0.003	1.58 (0.96–2.60)	0.070	1.40 (0.80–2.47)	0.239	1.42 (0.79–2.55)	0.243
Cigarette smoking (yes vs. no)	1.97 (1.43–2.70)	<0.001	1.09 (0.75–1.58)	0.667	1.62 (1.14–2.29)	0.007	0.93 (0.62–1.39)	0.711
Betel quid chewing (yes vs. no)	1.74 (1.37–2.20)	<0.001	1.14 (0.86–1.50)	0.370	1.79 (1.35–2.36)	<0.001	1.30 (0.94–1.81)	0.109
Alcohol consumption (yes vs. no)	1.80 (1.42–2.29)	<0.001	1.25 (0.96–1.62)	0.094	2.05 (1.55–2.71)	<0.001	1.55 (1.14–2.11)	0.005
Presence of medical comorbidities	1.47 (1.16–1.87)	0.001	1.31 (1.03–1.68)	0.031	1.23 (0.94–1.62)	0.134		
Chemotherapy (yes vs. no)	0.79 (0.57–1.09)	0.144			0.72 (0.50–1.04)	0.079		
RT technique (IMRT vs. VMAT)	1.53 (1.21–1.95)	<0.001	1.54 (1.20–1.97)	<0.001	1.59 (1.20–2.10)	0.001	1.54 (1.16–2.05)	0.003
Treatment duration (>56 vs. ≤56)	1.36 (1.02–1.80)	0.035	1.30 (0.97–1.75)	0.077	1.20 (0.85–1.68)	0.292		
EQD2 ^b^	0.99 (0.93–1.06)	0.840			0.98 (0.90–1.06)	0.587		

Abbreviations: AJCC, American Joint Committee on Cancer; CI, confidence interval; EQD2, equivalent dose in 2 Gy fractions; HR, hazard ratio; OS, overall survival; PFS, progression-free survival; PIV-6, pan-immune–inflammation value at six weeks of treatment; PIV-4, pan-immune–inflammation value at four weeks of treatment; PIV-2, pan-immune–inflammation value at two weeks of treatment; PIV-0, pretreatment pan-immune–inflammation value; RT, radiotherapy; IMRT, intensity-modulated radiation therapy; VMAT, volumetric modulated arc therapy. ^a^ Dichotomized according to the optimal cut-off value determined by ROC curve. ^b^ Continuous variable. ^c^ All significant factors (*p* < 0.05) in the univariable analysis were entered as covariates in the Cox multivariable regression analysis using the Enter method.

## Data Availability

The datasets used and analyzed during the current study are available from the corresponding author upon reasonable request.

## Supplementary Materials

The following supporting information can be downloaded at: https://www.mdpi.com/article/10.3390/biomedicines14040830/s1. Figure S1: CONSORT-style Flow Diagram of Selection Process; Figure S2: Schoenfeld Residual Plots for the OS and PFS; Figure S3: Kaplan-Meier plots of OS and PFS according to PIV ratios at two weeks (A–B), four weeks (C–D), and six weeks (E–F) after the start of radiotherapy in patients with nasopharyngeal carcinoma (*n* = 338); Figure S4: Kaplan-Meier plots of OS and PFS according to PIV ratios at two weeks (A–B), four weeks (C–D), and six weeks (E–F) after the start of radiotherapy in patients with oropharyngeal carcinoma (*n* = 156); Figure S5: Kaplan-Meier plots of OS and PFS according to PIV ratios at two weeks (A–B), four weeks (C–D), and six weeks (E–F) after the start of radiotherapy in patients with T3–4 disease (*n* = 389); Figure S6: Kaplan-Meier plots of OS and PFS according to PIV ratios at two weeks (A–B), four weeks (C–D), and six weeks (E–F) after the start of radiotherapy in patients with N2–3 disease (*n* = 356); Table S1: Chemotherapy strategy stratified by primary tumor sites; Table S2: Baseline characteristics of Nasopharyngeal carcinoma subgroup (*n* = 338).

## Abbreviations

The following abbreviations are used in this manuscript:

AJCC	American Joint Committee on Cancer
CCRT	Concurrent chemoradiotherapy
CBCs	Complete blood counts
CRC	Colorectal cancer
DAMP	Damage-associated molecular pattern
EQD2	Equivalent dose in 2 Gy fractions
HNC	Head and neck cancer
HRs	Hazard ratios
HMGB 1	High mobility group box 1
IQRs	Interquartile ranges
LMR	Lymphocyte-to-monocyte ratio
NSCLC	Non-small-cell lung cancer
NPC	Nasopharyngeal carcinoma
OS	Overall survival
PFS	Progression-free survival
PIV	Pan-immune–inflammation value
PD-1	Programmed Death 1
PD-L1	Programmed Cell Death-Ligand 1
ROC	Receiver operating characteristic
RT	Radiotherapy
Treg	Regulatory T-cells
