# Supplementary material for: Longitudinal Monitoring of Pan-Immune–Inflammation Value Forecast Outcomes for Patients with Head and Neck Cancer Treated with Chemoradiotherapy or Radiotherapy: Results from a Large Cohort Study
Source: Biomedicines. 2026 Apr 5;14(4):830. doi: 10.3390/biomedicines14040830 (PMC13113991; doi:10.3390/biomedicines14040830)
Supplement: Supplementary file 1 [file biomedicines-14-00830-s001.zip › biomedicines-4195601-supplementary.pdf]

Supplement Files

Supplement Figure S1. CONSORT-style Flow Diagram of Selection Process

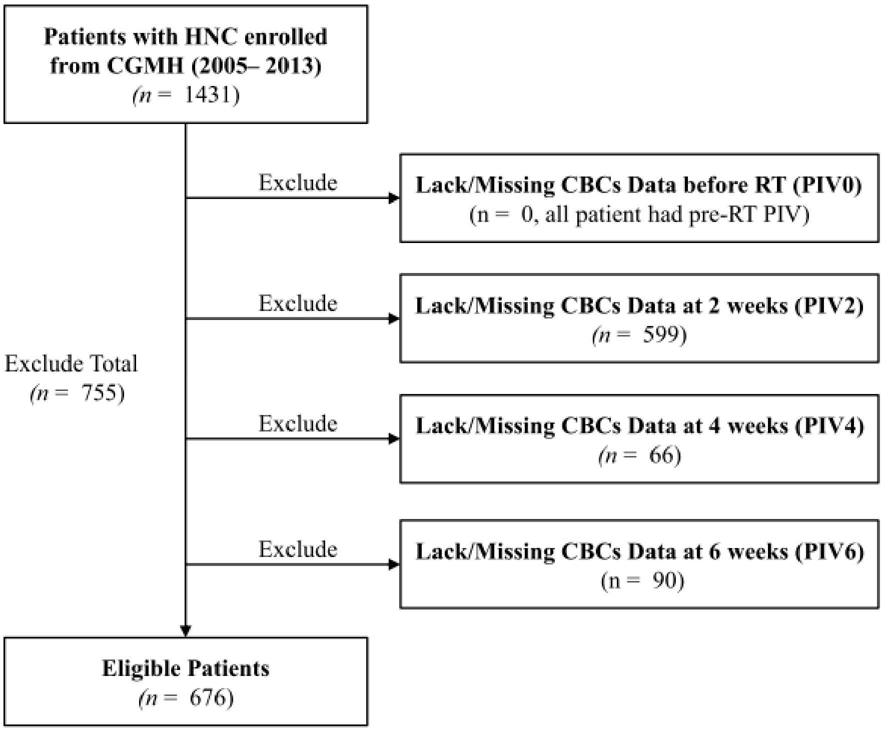

Supplement Figure S2. Schoenfeld Residual Plots for the OS and PFS

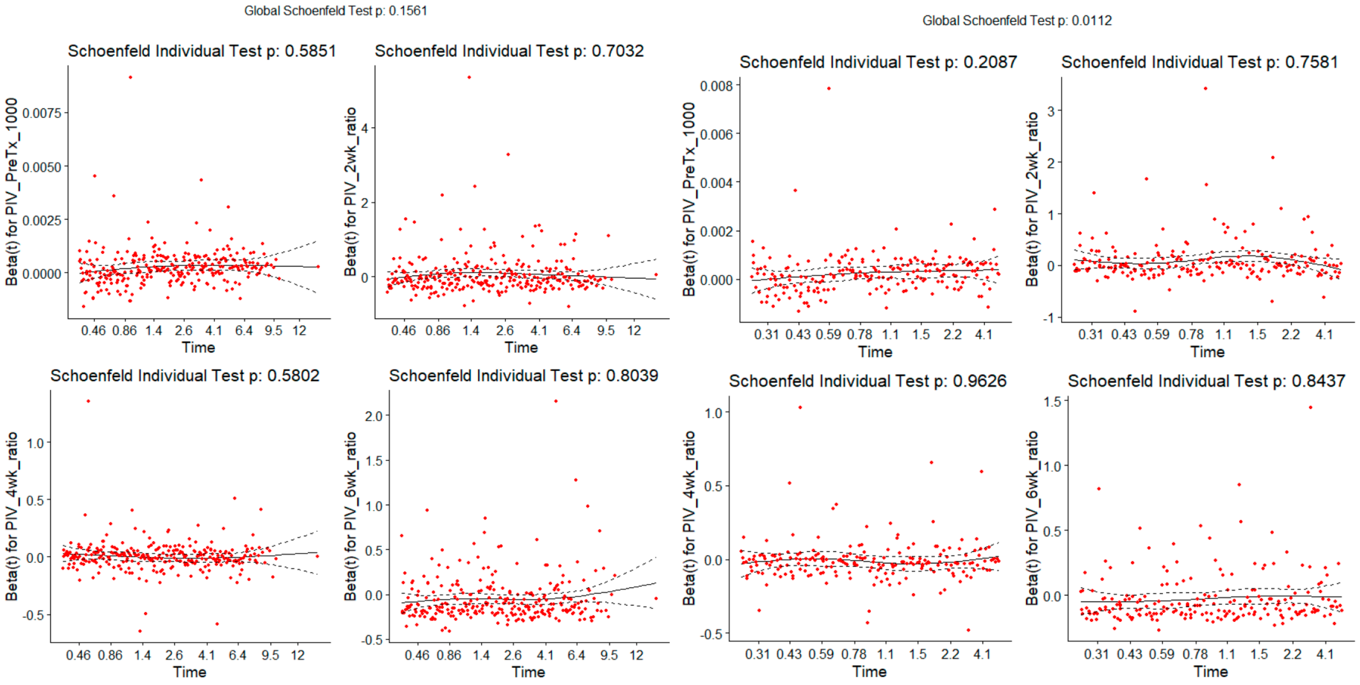

Supplement Figure S3. Kaplan-Meier plots of OS and PFS according to PIV ratios at two weeks (A–B), four weeks (C–D), and six weeks (E–F) after the start of radiotherapy in patients with nasopharyngeal carcinoma (n = 338).

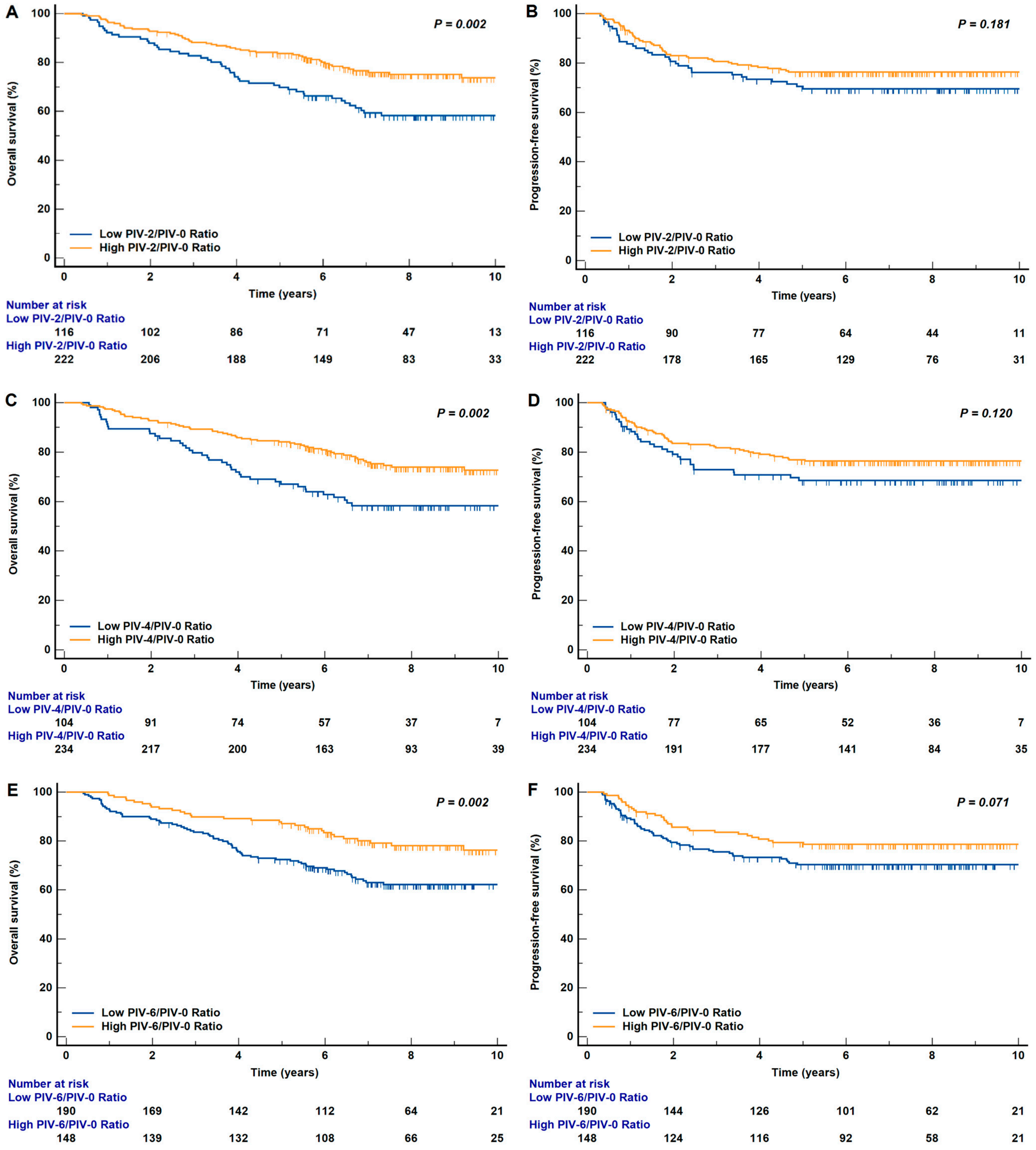

Supplement Figure S4. Kaplan-Meier plots of OS and PFS according to PIV ratios at two weeks (A–B), four weeks (C–D), and six weeks (E–F) after the start of radiotherapy in patients with oropharyngeal carcinoma (n = 156).

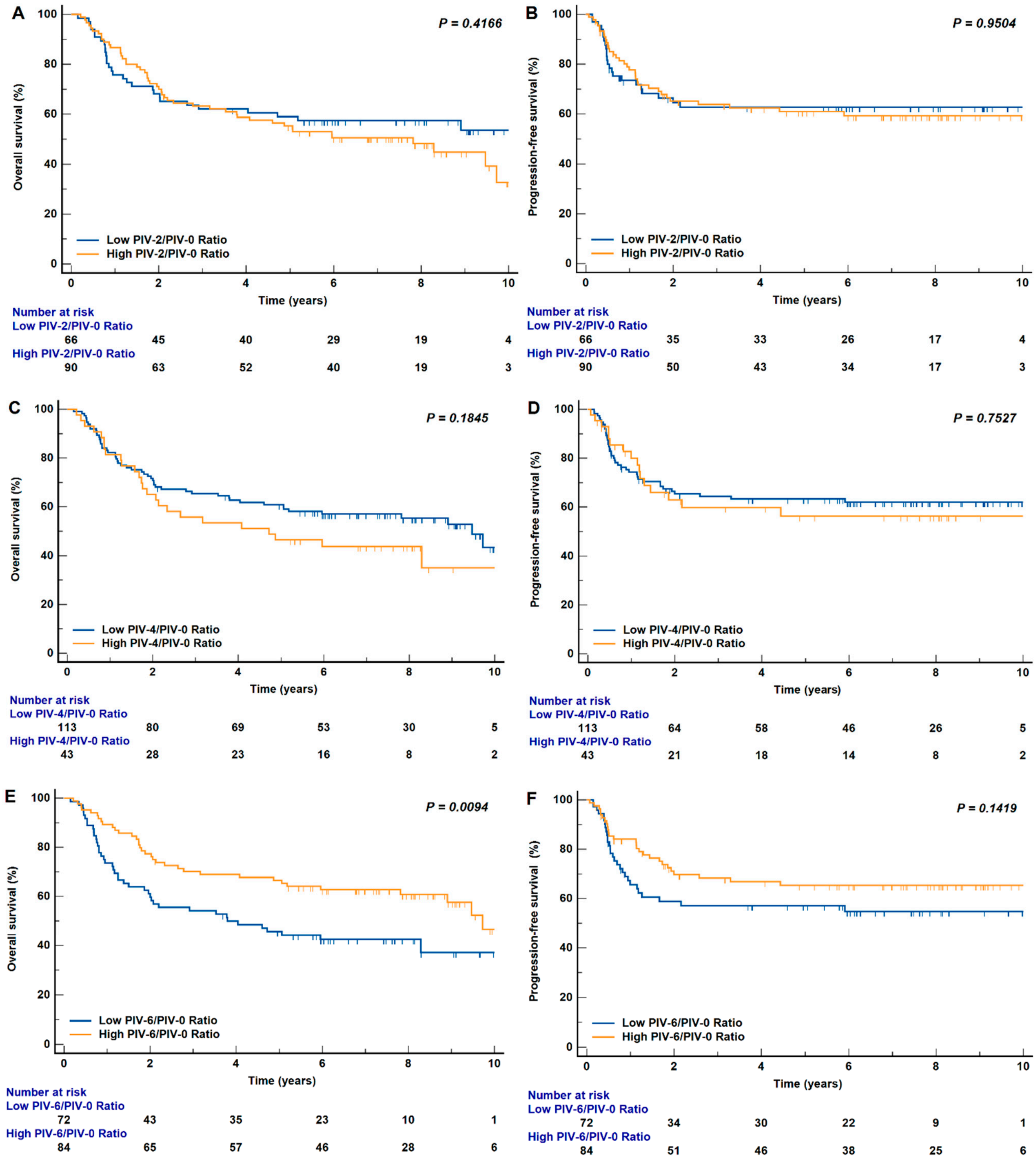

Supplement Figure S5. Kaplan-Meier plots of OS and PFS according to PIV ratios at two weeks (A–B), four weeks (C–D), and six weeks (E–F) after the start of radiotherapy in patients with T3–4 disease (n = 389)

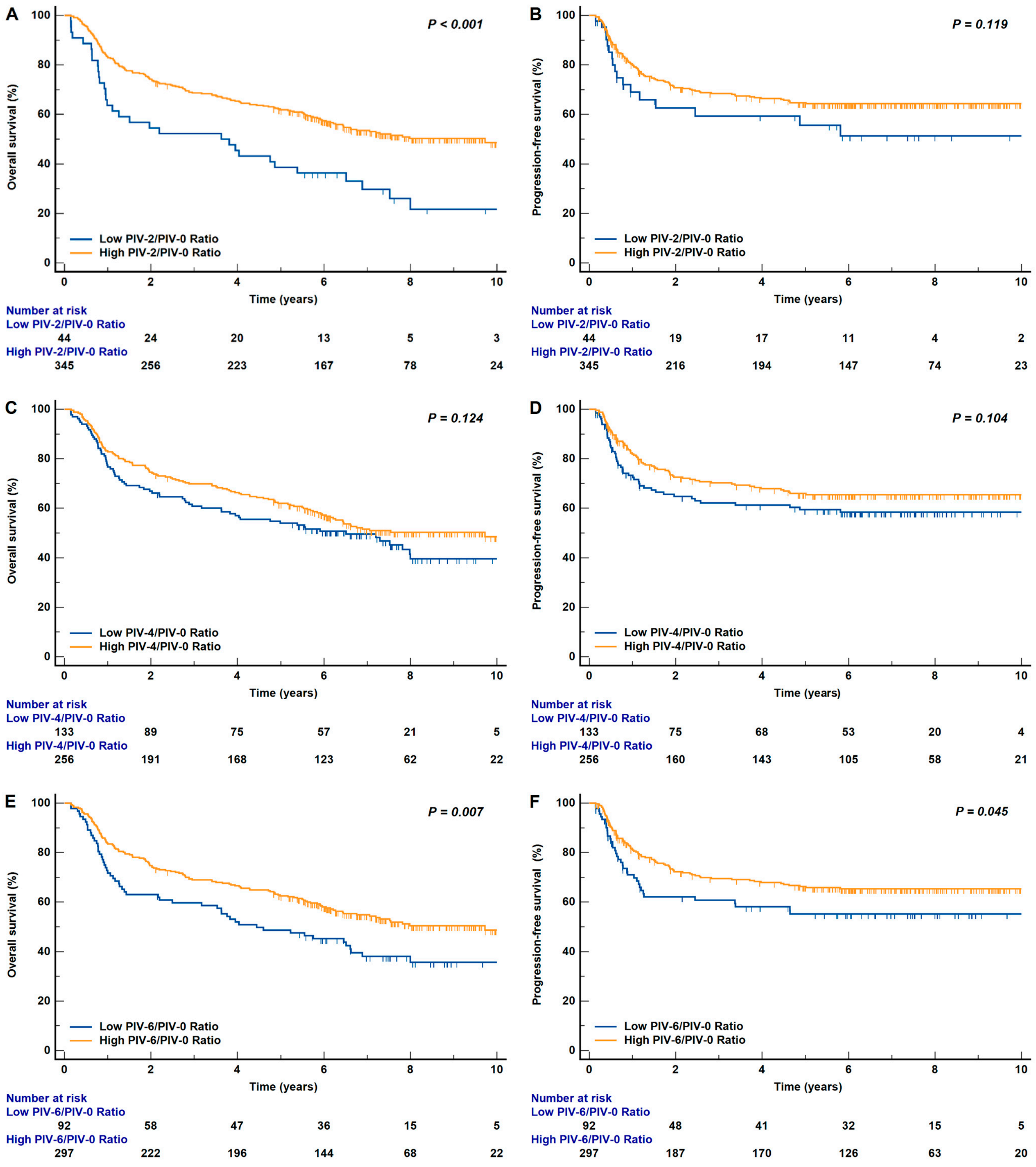

Supplement Figure S6. Kaplan-Meier plots of OS and PFS according to PIV ratios at two weeks (A–B), four weeks (C–D), and six weeks (E–F) after the start of radiotherapy in patients with N2–3 disease (n = 356).

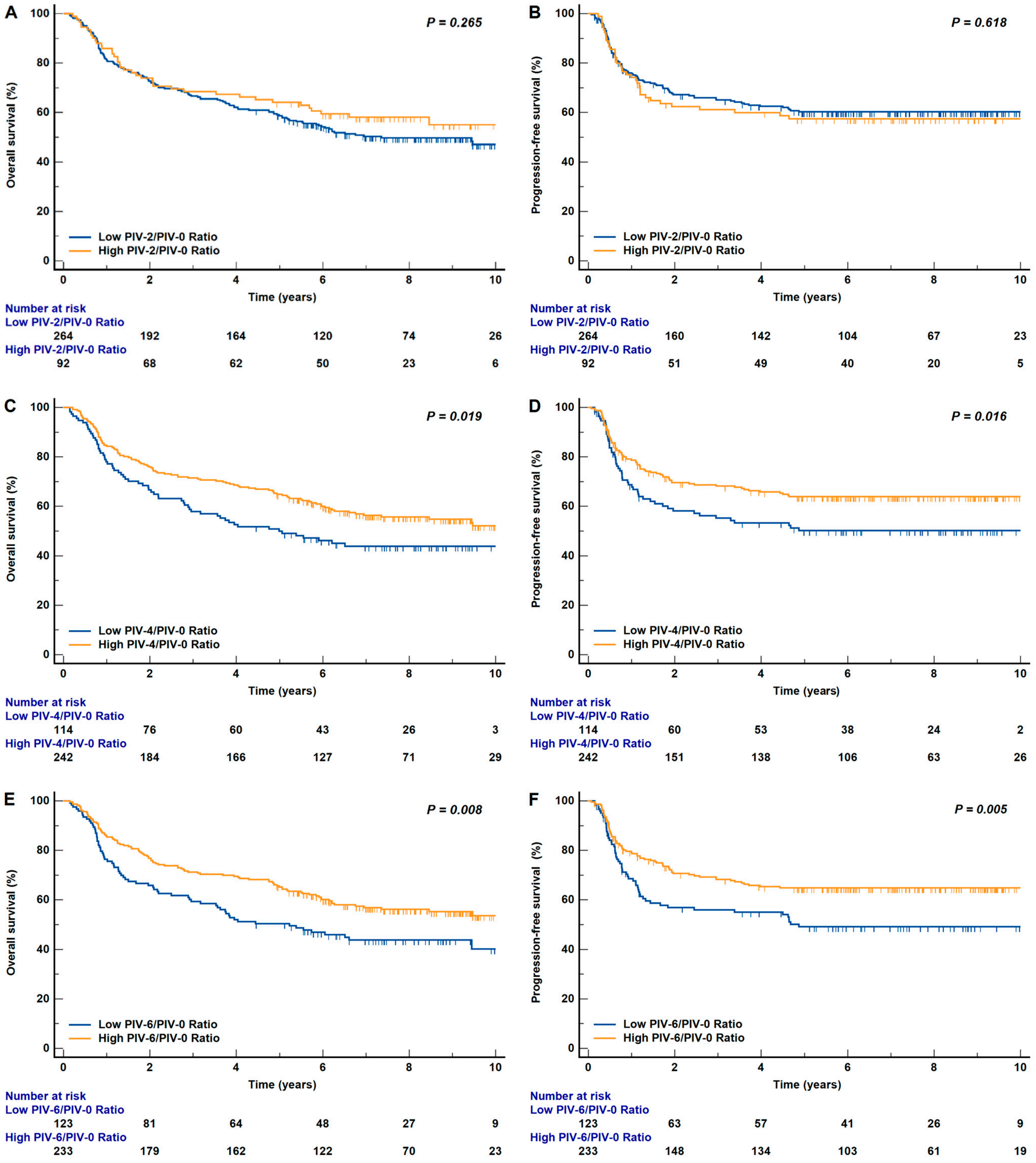

Supplement Table S1. Chemotherapy strategy stratified by primary tumor sites.

Variable	All Patients (n = 676)	Nasopharyngeal (n = 338)	Oropharynx (n = 156)	Hypopharynx (n = 136)	Larynx (n = 46)
Chemotherapy					
Yes	575 (85.1%)	321 (93.8%)	119 (76.3%)	104 (76.5%)	31 (67.4%)
No	101 (14.9%)	17 (6.2%)	37 (23.7%)	32 (23.5%)	15 (32.6%)
Regimen					
Cisplatin-based	552 (96.0%)	317 (93.8%)	111 (93.3%)	100 (96.2%)	24 (77.4%)
Carboplatin-based	6 (1.0%)	1 (0.3%)	3 (2.5%)	2 (1.9%)	0 (0%)
Taxotere-based	1 (0.2%)	1 (0.3%)	0 (0%)	0 (0%)	0 (0%)
Cetuximab-based	15 (2.6%)	1 (0.3%)	5 (4.2%)	2(1.9%)	7 (22.6%)
Others [a]	1 (0.2%)	1 (0.3%)	0 (0%)	0 (0%)	0 (0%)
Scheme					
Weekly platinum (30–40 mg/m²)	120 (20.9%)	88 (27.4%)	15 (12.6%)	14 (13.5%)	3 (9.6%)
Bi-weekly platinum (50 mg/m²) + Oral Tegafur +/- Leucovorin	438 (76.2%)	230 (71.7%)	99 (83.2%)	88 (84.6%)	21 (67.7%)
Others	17 (2.9%)	3 (0.9%)	5 (4.2%)	2 (1.9%)	7 (22.6%)

[a] Others chemotherapy regimen includes Taxotere, Cyclophosphamide, Epirubicin, 5-FU, or Cetuximab.

Abbreviations: 5-FU, 5-Fluorouracil.

Supplement Table S2. Baseline characteristics of Nasopharyngeal carcinoma subgroup (*n* = 338).

Characteristics	Value	Percentage
Median age, years (IQR)	48.1 (41.7–54.3)	
Gender		
Male	267	79.0
Female	71	21.0
AJCC 7th edition T stage		
T1–2	165	48.8
T3–4	173	51.2
AJCC 7th edition N stage		
N0–1	175	51.8
N2–3	163	48.2
WHO Stage		
I	12	3.6
II	18	5.3
III	308	91.1
Initial EBV DNA Quantitative PCR		
Available	178	52.7
Missing Data	160	47.3
Median EBV DNA Viral Load (copies/mL)	428.5 (72.0–2335.0)	
Radiation Therapy Technique		
IMRT	162	47.9
VMAT	176	52.1
Chemotherapy Regimen		
Cisplatin-based	317	93.8
Carboplatin-based	1	0.3
Taxotere-based	1	0.3
Cyclophosphamide, Epirubicin, and 5FU	1	0.3
Cetuximab	1	0.3
No chemotherapy	17	5.0

Abbreviations: AJCC, American Joint Committee on Cancer; EBV, Epstein-Barr Virus; IQR, interquartile range; IMRT, Intensity Modulated Radiation Therapy; VMAT, Volumetric modulated arc therapy; PCR, Polymerase chain reaction.
